# CodonTest: Modeling Amino Acid Substitution Preferences in Coding Sequences

**DOI:** 10.1371/journal.pcbi.1000885

**Published:** 2010-08-19

**Authors:** Wayne Delport, Konrad Scheffler, Gordon Botha, Mike B. Gravenor, Spencer V. Muse, Sergei L. Kosakovsky Pond

**Affiliations:** 1 Department of Pathology, University of California, San Diego, La Jolla, California, United States of America; 2 Computer Science Division, Department of Mathematical Sciences, Stellenbosch University, Stellenbosch, South Africa; 3 School of Medicine, University of Swansea, Swansea, United Kingdom; 4 Department of Statistics, North Carolina State University, Raleigh, North Carolina, United States of America; 5 Department of Medicine, University of California, San Diego, La Jolla, California, United States of America; University of Chicago, United States of America

## Abstract

Codon models of evolution have facilitated the interpretation of selective forces operating on genomes. These models, however, assume a single rate of non-synonymous substitution irrespective of the nature of amino acids being exchanged. Recent developments have shown that models which allow for amino acid pairs to have independent rates of substitution offer improved fit over single rate models. However, these approaches have been limited by the necessity for large alignments in their estimation. An alternative approach is to assume that substitution rates between amino acid pairs can be subdivided into 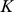 rate classes, dependent on the information content of the alignment. However, given the combinatorially large number of such models, an efficient model search strategy is needed. Here we develop a Genetic Algorithm (GA) method for the estimation of such models. A GA is used to assign amino acid substitution pairs to a series of 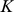 rate classes, where 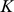 is estimated from the alignment. Other parameters of the phylogenetic Markov model, including substitution rates, character frequencies and branch lengths are estimated using standard maximum likelihood optimization procedures. We apply the GA to empirical alignments and show improved model fit over existing models of codon evolution. Our results suggest that current models are poor approximations of protein evolution and thus gene and organism specific multi-rate models that incorporate amino acid substitution biases are preferred. We further anticipate that the clustering of amino acid substitution rates into classes will be biologically informative, such that genes with similar functions exhibit similar clustering, and hence this clustering will be useful for the evolutionary fingerprinting of genes.

## Author Summary

Evolution in protein-coding DNA sequences can be modeled at three levels: nucleotides, amino acids or codons that encode the amino acids. Codon models incorporate nucleotide and amino acid information, and allow the estimation of the rate at which amino acids are replaced () versus the rate at which they are preserved (). The  ratio has been used in thousands of studies to detect molecular footprints of natural selection. A serious limitation of most codon models is the unrealistic assumption that all non-synonymous substitutions occur at the same rate. Indeed, amino acid models have consistently demonstrated that different residues are exchanged more or less frequently, depending on incompletely understood factors. We derive and validate a computational approach for inferring codon models which combine the power to investigate natural selection with data-driven amino acid substitution biases from alignments. The addition of amino acid properties can lead to more powerful and accurate methods for studying natural selection and the evolutionary history of protein-coding sequences. The pattern of amino acid substitutions specific to a given alignment can be used to compare and contrast the evolutionary properties of different genes, providing an evolutionary analog to protein family comparisons.

## Introduction

Modern molecular evolution has benefited greatly from the development of a sound probabilistic framework for modeling the evolution of homologous gene sequences [1]. In particular, codon substitution models [2], [3] have facilitated the estimation of the ratio of non-synonymous to synonymous substitution rates (referred to as 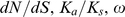), which can be interpreted as an indicator of the strength and type of natural selection (see [4] or [5] for recent reviews). Codon models are fundamentally mechanistic because they use the structure of the genetic code to partition codon substitutions into classes. Initially, and in most subsequent applications of codon models, all one-nucleotide substitutions were stratified into synonymous (rate 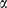, using the notation of [2]) and non-synonymous (rate 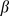) classes. Despite several early attempts, e.g. [3], none of the widely-adopted codon models incorporated physicochemical properties of the two residues being exchanged. In contrast, most protein substitution models are derived by estimating the relative rates of amino-acid substitutions in large protein databases [6]–[8], and consistently report dramatic differences in the relative replacement rates of different residues.

The persisting dissonance between how codon and protein models approach amino acid substitution rates has fostered multiple recent efforts to develop what we will call *multi-rate* codon models (or more accurately, multi- nonsynonymous rate models), in contrast to the existing single-rate model. These models divide amino acid pairs (or codon pairs) into multiple rate categories, such that every category has its own rate which governs substitutions between the pairs in that category. In the most extreme case, every amino acid or codon pair belongs to a different category and thus has its own rate – potentially leading to a very large number of parameters that need to be estimated. Several strategies have been proposed for limiting the number of parameters in multi-rate models.

Doron-Faigenboim et al. [9] proposed to overlay existing empirically derived amino acid substitution matrices (e.g. [7] or [8]) onto single-rate codon models by weighted partitioning of the empirical rate of substitution between two protein residues. Kosiol, Holmes & Goldman [10] directly estimated all 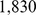 codon-to-codon substitution rates in an empirical codon model – a codon equivalent of the nucleotide GTR model [11], assuming the universal genetic code. However, this effort required a truly massive training dataset encompassing alignments from 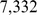 protein families of the Pandit database [12]. The resulting empirical codon model (ECM) encodes evolution patterns averaged over many proteins. However, no single empirically-derived substitution rate matrix appears to be generalizable across multiple genes and taxonomic groups, as evidenced by a plethora of specialized substitution models, e.g. for mammalian mitochondrial genomes [13], plant chloroplast genes [14], viral reverse transcriptases [15] or HIV-1 genes [16].

More mechanistic parameters can be introduced to improve biological realism of codon-models. The linear combination of amino acid properties (LCAP) model [17] expresses exchangeability of a pair of codons as an (exponentiated) linear combination of differences in five independently validated amino acid physicochemical properties. This parameterization incorporates weighting (or importance) coefficients inferred from the data to allow for differences in protein evolution between genes, shown to be significant and biologically meaningful in yeast proteins [18], and once again underscoring the utility of gene-specific evolutionary models.

All multi-rate codon models published to date have shown clear improvements in model fit over the single-rate model. However, multi-rate models in which substitutions were randomly assigned to classes easily outperform the single-rate model [19] and thus it is a poor performance benchmark. At the other extreme of model space is the full time-reversible codon model, with 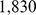 parameters (or 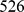, if only single nucleotide substitutions are modeled), which will certainly suffer from massive over-fitting on single gene alignments. Over-parameterization can be reduced by “smoothing”, i.e. by grouping the rates into exchangeability classes based on the physicochemical properties of amino acids [20]. However, without a rigorous model selection framework, it is difficult to ascertain how well any particular smoothing approach fits the data. To appreciate how large the space of potential models is, consider that there are are approximately 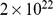 possible multi-rate codon models with 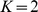 nonsynonymous rate classes, and approximately 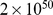 possible models for 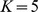. Given such a large search space it is impossible to evaluate even a small fraction of possible models exhaustively, and one cannot presume that any given model or a small set of models are sufficiently representative without exploring the alternatives.

Huelsenbeck et al. [21] examined a Bayesian approach to estimate empirical amino acid substitution models in which amino acid exchangeability classes are assigned using a Dirichlet process. However, a prior distribution needs to be specified for the number of classes (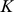 = 2, 5, or 10), and mechanistic features of codon evolution are excluded. Models which combine empirical codon models and mechanistic parameters, such as 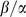 and transition-transversion bias [10], have been shown to outperform the models which include only a single effect. This evidence highlights the necessity to model both mutational effects, which result in substitution preferences for particular amino acids, and selective effects, the result of fitness differences of alternate phenotypes. In this manuscript, we present an information-theoretic model selection procedure that extends the concept of ModelTest [22], formulated for nucleotide model selection, to codon models. Unlike ModelTest, which examines 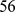 *a priori* defined models, we use a Genetic Algorithm (GA) to search the combinatorially large set of codon models (i.e. select the number of rate classes), to assign amino acid substitution rates to these classes, infer rate parameters and, finally, report a set of credible models given the data. Our group has successfully applied GAs to a variety of problems in evolutionary biology, including inference of lineage-specific selective regimes [23], detecting recombination in homologous sequence alignments [24], and model selection for paired RNA sequences [25], where the GA was able to recover biologically relevant properties and outperformed all known mechanistic models.

Using simulated data, we demonstrate that GA model selection (under a sufficiently stringent model selection criterion) is not susceptible to over-fitting, and that codon alignments of typical size contains sufficient signal to reliably allocate non-synonymous substitutions into a small number of rate classes, typically 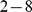. On empirical data sets, GA-selected codon substitution models consistently outperformed published empirical and mechanistic models. In addition to selecting a single best fitting model, the GA also estimates a set of credible models for an alignment. A weighted combination of models in the credible set enable model averaged phylogenetic [26] and substitution rate matrix [25] inference and further reduces the risk of over-fitting. We anticipate that improvements in model realism will translate into improved sequence alignment, phylogeny estimation, and selection detection. Moreover, we hypothesize that the clustering of non-synonymous substitution rates into groups with the same rate parameter is shared by genes with similar biological and structural properties, and hence this clustering is informative for improving evolutionary fingerprinting of genes [27].

## Methods

### Model definition

Models considered in this paper assume that codon substitutions along a branch in a phylogenetic tree can be described by an appropriately parameterized continuous-time homogeneous and stationary Markov process; an assumption ubiquitous in codon-evolution literature. The substitution process is uniquely defined by the rate matrix, 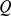, whose elements 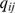 denote the instantaneous substitution rate from codon 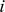 to codon 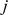. Using 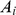 to label the amino-acid encoded by codon 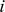, and assuming a universal genetic code with three stop codons (other codes can be handled with obvious modifications), matrix 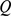 comprises 61×61 such elements, where
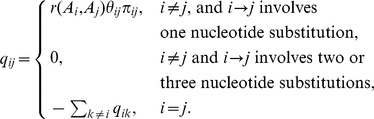
(1)Here, 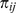 denote equilibrium frequency parameters, 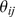 denote nucleotide mutational biases, and 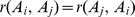 denote the substitution rates between amino acids encoded by codons 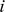 and 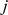. How to infer 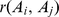 is the primary focus of this paper. We consider two different parameterizations of 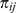: the GY parameterization [3], where 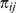 is the equilibrium frequency of the target *codon*, and the MG parameterization [2], where 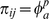 is a *nucleotide* frequency parameter for the position that is being substituted (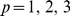; 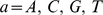). For the GY parameterization, we estimate codon equilibrium frequencies by their proportions in the data (the F61 estimator, 60 parameters for the universal genetic code). For the MG parameterization, we estimate the nine frequency parameters by maximum likelihood [28]. The equilibrium frequency of codon 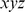 can then be computed as
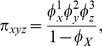
where 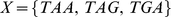 and 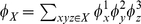.

Finally, we set 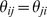, 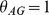 and estimate 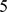 other rates (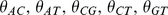) by maximum likelihood; this parameterization follows the MG94
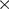
REV model from [29].

### Inferring non-synonymous substitution rates

By varying the parametric complexity of the non-synonymous substitution rate 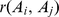 encoding in equation (1), we can span the range of models from the single rate model (SR, current default standard, 1 non-synonymous rate parameter), to the general codon time-reversible model (REV) with each amino-acid pair substitution exchanged at its own rate. Only 75 out of 190 total amino-acid pairs can be exchanged via a single nucleotide substitution, for example 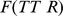 and 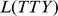 are one such pair, but 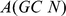 and 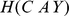 are not. Consequently, the REV model has 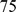 non-synonymous rate parameters. The purpose of our study is to explore the model space between these two extremes, taking into account the limitations of information content in single gene alignments. Note that most existing multi-rate models can be represented with an appropriate choice of 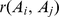 in equation (1). Empirical models (e.g. ECM) replace 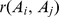 with numerical values estimated from large training data sets, whereas mechanistic models (e.g. LCAP) assume that rates can be modeled via a function measuring differences/similarities in physicochemical properties of residues (Table 1).

**Table 1:** Various approaches to estimating residue-dependent non-synonymous substitution rates.

Model	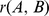	p	Description
Single rate	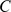	1	
Random – X	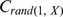	X	Rates randomly assigned to 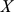 classes
ECM	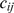	0	Codon level rates 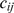 are inferred from a large training data set
ECM+ 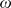	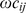	1	Codon level rates 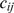 are inferred from a large training data set
			Correction parameter 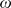 inferred from the data
LCAP	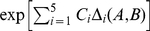	5	Based on a weighted combination of 5 physico-chemical distances 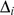
GA - X	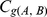	X	X and 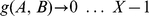 are inferred by the GA
REV	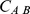	75	Each unique residue pair within one nucleotide substitution has its own rate

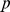
 = number of model parameters estimated from the data. 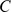 denotes rates that are estimated by maximum likelihood by the data and 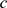 – those that are estimated in other ways.

We focus on structured (or rate clustering) models: those which assume that substitution rates can be partitioned/structured into 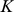 classes, where each class has a single estimated rate parameter. These structured models may be defined using amino acid similarity classes [30], but instead of adopting *a priori* classes of rates, we propose to *infer* their number and identity from the data. A structured model with 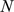 substitutions (e.g. 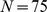 for the Universal genetic code) in 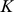 classes can be represented as a vector 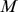 of length 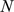, where each element is an integer between 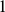 and 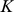 labeling the class. For example if the vector entries corresponding to 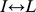, 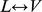 and 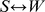 substitutions have values 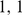 and 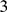, then 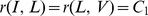 and 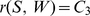. As an analogy, the HKY85 nucleotide model [31] is a structured model with vector, 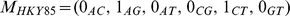, where the substitutions between 6 nucleotide pairs (indicated by a subscript) are placed into transition (1) and transversion (0) classes. Given the structure of a codon model, e.g. 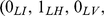 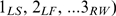, it can be fitted to the data using standard maximum likelihood phylogenetic algorithms, e.g. as implemented in HyPhy [32]. The resulting set of rate estimates 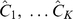 instantiate a structured model and induce a corresponding empirical model, e.g. 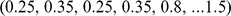.

Because the space of structured codon models is combinatorially large, we utilize a GA previously used to solve an analogous model selection problem for paired RNA data [25]. Parameter space is defined by two components: a discrete component which assigns pairwise non-synonymous substitutions between codons to 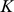 rate classes using the structured vector described above, and a continuous component comprising a vector of branch lengths, nucleotide substitution rates, frequency parameters and non-synonymous rates 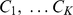. The discrete component is optimized by the GA, while the continuous component is estimated using numerical non-linear optimization procedures, given the structure of the model. We initially approximate branch lengths using the SR model and update them whenever the GA iteration improves the fitness score by more than 50 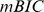 points (see below) as compared to the most recent model for which branch lengths have been estimated. Further details of the genetic algorithm are described in detail in [25], and for the sake of brevity we do not present it here.

We are left with the problem of inferring the number of rate classes 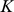. This is done by starting with 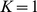 and iteratively proposing to increment 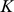. For each proposal, the model with 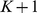 rate classes is optimized using the optimized 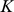-class model as initialization. If the proposal results in a model with a better fitness value (see below), it is accepted and a new proposal generated. The process terminates when the 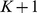-class proposal does not beat the 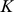-class model.

**Figure 1:**
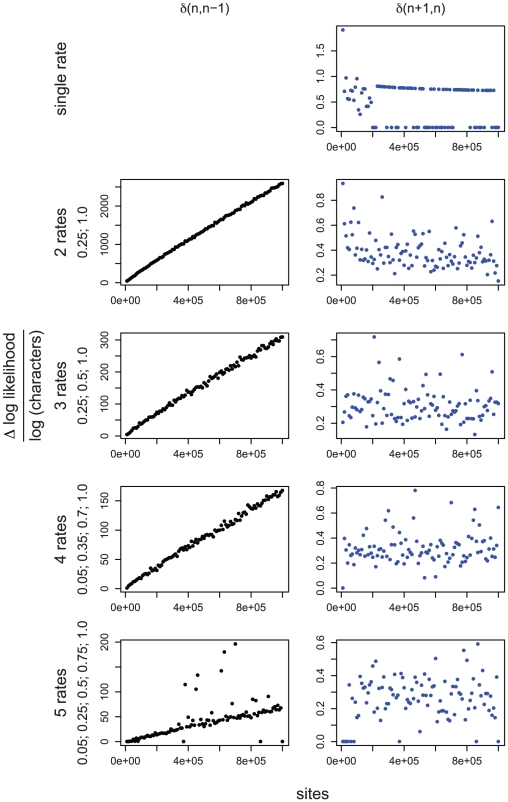
Simulation studies used to derive the appropriate penalty term for 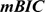. Each panel plots the difference in log likelihood (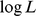) normalized by the logarithm of the sample size (number of characters), between best fitting GA models with 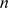 and 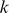 rates (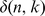), against the number of sites in the alignment. For simulations with a single rate class we plotted 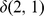, top right. Figures for multiple rate simulations (2–5 rates) show 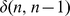 as black dots (left column); and 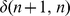 as blue dots (right column). Values to the right of row report simulated rates for each class. The left column is a reflection of power, whereas the right column – of the degree of over-fitting. For the case where a single rate was simulated, the degree of over-fitting is the rate of false positives. The desired behavior for 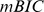 is achieved when the model with 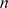 rate classes is preferred to models with 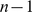, and 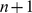 rate classes. For a modified BIC criterion 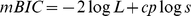 with 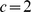, the former happens if 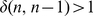 (more definitively with increasing sample size), and the latter if 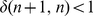 (regardless of sample size).

We initially assigned a fitness value to each model using 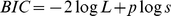 where 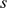 is the sample size and 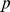 is the number of parameters in the model [33]. The “sample size” of a sequence alignment is difficult to quantify with a single number, since it depends on both the number of sequences in the alignment and the lengths of those sequences. We use the number of characters to approximate “sample size” to make the model selection criterion maximally conservative. While it is straightforward to count the number of estimated parameters in any given structured model, setting 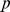 to that number leads to model over-fitting (results not shown), because the topological component (the assignment of rates to classes) adds further “degrees of freedom” to the model. To determine the appropriate penalty term, we conducted simulations; there is precedent for this in statistical literature on generalized information criteria (e.g. [34]). We removed the effect of phylogeny by simulating nine sets of two-sequence alignments (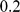 divergence): each set of simulations consisted of 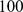 replicates with between 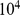 and 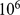 codons (in 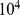 increments). The sets had 1 to 5 rate classes (Figure 1), representing rate classification problems that ranged from easy (large numerical differences between class rates, e.g. 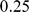 and 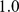) to difficult (small numerical differences, e.g. 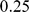 and 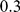). We constructed generating multi-rate models by assigning rates to 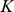 bins randomly with equal probability. For each simulation set we plotted the difference in log likelihood (scaled by the sample size = log of characters) between the correct model (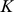 rates), and models with 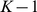 and 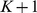 rates, respectively. Simulations indicated that doubling the number of parameters in the BIC penalty term ensured sufficient power, and controlled false positives for all simulation sets (Figure 1). We used this modified BIC, 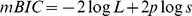 to assign fitness to every model examined by a GA run and select those with the lowest 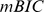.

### Simulated data analysis

We also simulated realistic “gene-size” alignments on 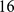 and 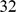 taxon trees. Nucleotide frequencies were uniform (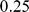) for each position, and the nucleotide bias component was set to HKY85 with transition/transversion ratio, 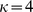. We generated 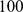 data sets for each 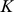:rate vector combination, under the single rate, and a fixed Random-K model (Table 2). These data allowed us to assess the performance of the model when the true underlying model was known.

**Table 2:** The performance of GA model selection with 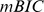 in estimating the number and membership of 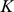 rate classes as well as rate values from simulated data.

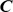	taxa	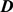	simulated rates	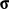	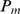	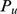	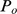	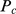
1	2	0.2	n/a	n/a	0.99	n/a	0.01	n/a
2	2	0.2	(0.25, 1.0)	(0.004, 0.010)	1.00	0	0	1.00
			(0.25, 0.3)	(0.012, 0.009)	0.98	0.02	0	0.860
3	2	0.2	(0.25, 0.5, 1.0)	(0.011, 0.015, 0.053)	1.00	0	0	0.996
			(0.25, 0.35, 0.5)	(0.004, 0.011, 0.008)	0.97	0.03	0	0.971
4	2	0.2	(0.05, 0.35, 0.7, 1.0)	(0.006, 0.021, 0.040, 0.041)	0.99	0.01	0	0.993
			(0.5, 0.65, 0.75, 1.0)	(0.004, 0.007, 0.006, 0.006)	0.82	0.18	0	0.936
5	2	0.2	(0.05, 0.25, 0.5, 0.75, 1.0)	(0.003, 0.012, 0.008, 0.014, 0.012)	0.91	0.09	0	0.981
			(0.5, 0.65, 0.75, 0.85, 1.0)	(0.003, 0.005, 0.006, 0.007, 0.010)	0.67	0.33	0	0.927
1	16	0.2	n/a	n/a	1.00	0	0	n/a
2	16	0.2	(0.25, 1.0)	(0.016, 0.044)	1.00	0	0	0.923
3	16	0.2	(0.25, 0.5, 1.0)	(0.022, 0.045, 0.052)	0.23	0.77	0	0.713
		0.2	(0.25, 0.75, 1.5)	(0.019, 0.050, 0.061)	1.00	0	0	0.837
		0.5	(0.25, 0.5, 1.0)	(0.014, 0.022, 0.037)	1.00	0	0	0.861
3	32	0.2	(0.25, 0.5, 1.0)	(0.018, 0.026, 0.038)	0.89	0.11	0	0.817

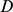
 measures the simulated pairwise sequence divergence (expected substitutions/nucleotide site); 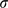, standard deviation (averaged over replicates) of estimated rates from the generating values; 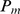, the proportion of simulations for which the correct number of rate classes are inferred; 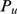, the proportion of simulations which are under-fitted, 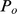, the proportion of simulations which are over-fitted, and 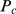, the mean Rand C-statistic [35] between rate clusters in the generating model and that in the inferred models.

For each simulation scenario, we report the proportion of replicates 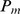 for which the GA inferred the correct number of rate classes 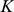, the proportion of underfitted replicates 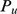 (too few rate classes were inferred) and the proportion of overfitted replicates 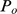 (too many rate classes were inferred). For the replicates where the correct number of rate classes was inferred, we computed the Rand statistic (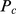, [35]) on the generating and inferred model structures to quantify the similarity between two clusterings rates. The Rand statistic quantifies the similarity between two clusterings (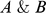) of the same set of 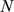 objects and can be defined as 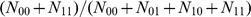, where 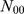 is the number of objects (pairs of substitution rates) that belong to different classes in both A and B, 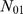 (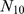) is the number of objects that belong to different (same) classes in A, but the same (different) class in B, and 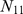 is the number of objects that belong to the same class in both A and B. Clearly, 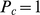 for perfect agreement (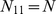) and 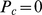 for perfect disagreement (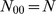).

### Empirical data analysis

We prepared a collection of reference empirical data sets (see Table 3), to be used for benchmarking GA, published and extreme-case models. The collection included three protein family alignments from Pandit [12] selected randomly from all alignments with 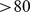 taxa, a randomly selected Yeast protein alignment [18], a group M HIV-1 *pol* alignment [36] and an Influenza A virus (IAV) *HA* alignment comprising H3N2, H5N1, H2N2 and H1N1 serotypes. The latter was assembled by random selection of 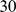 post-2005 sequences for each serotype from the NCBI Influenza database [37]. Finally, we examined the vertebrate rhodopsin protein, recently analyzed for molecular mechanisms of phenotypic adaptation by [38]. We inferred a structured multi-rate model for each of these data sets using the genetic algorithm and 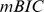 model fitness function defined above. A comparison of the GA-fitted model against existing models is unfair, since the former was selected among a set of candidate models using the test alignment. To confirm that GA models were generalizable, we evaluated the fit of the GA models and that of existing models for both the reference datasets, and independent test alignments for the same taxonomic groups (validation data sets). Two HIV-1 pol gene alignments were obtained for subtypes B [39] and C [40]. Subtype assignments were confirmed using the SCUEAL sub-typing tool [36], and inter- and intra-subtype recombinants were pruned from the analysis. For IAV *HA* we used independent alignments for serotypes H5N1 and H3N2, filtered from the NCBI Influenza database [37], and from [41], respectively.

**Table 3:** Empirical data set characteristics.

source	Taxon	Gene	# taxa	# sites	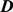	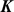	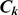
Pandit/Pfam (PF03477)*	Multiple	ATP cone	72	312	66.6†	5	(0.007, 0.036, 0.144, 0.341, 3.108)
Pandit/Pfam (PF06455)*	Multiple	NADH5 C	82	552	1.68	4	(0.043, 0.208, 0.456, 0.910)
Pandit/Pfam (PF02780)*	Multiple	Transketolase C	83	393	3.00	6	(0.002, 0.033, 0.094,
							0.268, 0.678, 4.744)
[38] *	Vertebrate	Rhodopsin	38	990	0.44	4	(0.018, 0.116, 0.371, 0.724)
[18]	Yeast	Pyruvate kinase	16	1389	0.51	4	(0.024, 0.093, 0.226, 0.608)
(YAL038W)*							
NCBI*	HIV-1 group M	*pol*	142	2847	0.15	7	(0.047, 0.114, 0.211, 0.350,
							0.532, 0.998, 1.562)
[39]	HIV-1 subtype B	*pol*	371	1497	0.06	n/a	n/a
[40]	HIV-1 subtype C	*pol*	348	1170	0.09	n/a	n/a
NCBI*	Seasonal IAV	*HA*	349	987	0.09	3	(0.350, 1.211, 3.287)
NCBI	IAV A H5N1	*HA*	279	1545	0.04	n/a	n/a
[41]	IAV A H3N2	*HA*	68	987	0.02	n/a	n/a

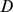
 is mean pairwise nucleotide divergence (substitutions/site, estimated under the single rate codon model), 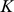 is the number of rates estimated in the GA, 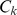 are the maximum likelihood estimates for the rates.

*Reference alignments for which GA models were estimated. All GA results presented are for the model with best 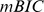.

**†:** ATP cone is comprised of highly divergent sequences, with only 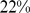 average pairwise amino-acid identity; synonymous rates appear to be saturated.

We fitted five reference models to each dataset: (i) the single-rate model, (ii) a Random-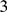 and a Random-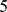 model, (iii) the empirical codon model (ECM, [10]), (iv) the Linear Combination of Amino Acid Properties (LCAP) model [17], [18], and (v) the reversible (REV) model (see Table 1).

For every dataset, the corresponding GA-run was processed to obtain three different alignment-specific multi-rate models.

1. A structured GA model (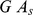): this is the best-fitting model (with value 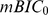), which defines 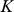 rate clusters. The numerical values of corresponding 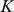 substitution rates are inferred using maximum likelihood. This model is a direct analog of the single “best” substitution model reported by the familiar ModelTest [22] nucleotide model selection procedure.
2. A numerical model-averaged GA model (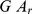), which is computed by weighting the numerical rate estimates from all models in the credible set using 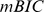-based Akaike weights (as in [25]). Briefly, for the 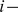
th model examined by the GA, we compute its evidence ratio versus the 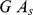 model as 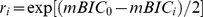, which can be thought of as the probability that model 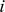 is the best model to explain the data, in the sense of minimizing the Kullback-Leibler divergence from the “true” unobserved model [42]. In addition to the 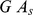 model, we also construct a set of credible models, i.e. all those models whose 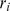 is sufficiently large (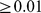). From this credible set we compute a model averaged estimate of any parameter 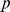, by a weighted sum of the estimate under model 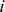, 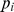 as 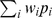, where the Akaike weight of model 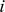, 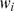 is defined as 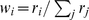. This 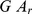 model is an analog of an empirical substitution model (e.g. ECM), and has no rate parameters that are estimated from validation data sets. By combining information from multiple models, statistical noise may be reduced (e.g. [26]).
3. The numerical 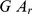 model with the addition of a single non-synonymous substitution rate parameter (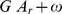) which multiplies all non-synonymous substitution rates in the 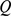 matrix. The direct analog is the 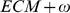 model of [10], and its purpose is to add a dataset specific “adjustment” to the baseline numerical model, since the estimated parameters of the baseline numerical model are weighted over the credible set and fixed at these estimates when applied to other datasets.

We used both BIC [33] and Likelihood ratio tests, where appropriate, for model comparison. These goodness-of-fit comparisons allowed us to evaluate whether a model estimated on reference alignments yielded a significant improvement over the other models when fitted to independent alignments for the same taxonomic groups. All models were implemented with the F61 frequency parameterization, in addition to their original frequency parameterizations, because the methodology used to estimate the ECM model precluded the use of other frequency parameterizations for across-the-board comparison. Alignments and phylogenetic trees were provided for the Pandit data set. In all other cases, alignments were generated using codon alignment tools implemented in HyPhy [32]. Maximum likelihood phylogenetic trees were estimated using PhyML [43] under a GTR [44] model of nucleotide substitution and among-site rate variation modeled as a discretized gamma distribution with 4 rate-classes [45]. Empirical alignments and trees are available at http://www.hyphy.org/pubs/cms/.

### Rate matrix comparisons

The entries of the substitution rate matrix 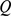 can be used to estimate the expected number of substitutions per site per unit time, 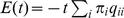, and to determine the value of the time parameter (assuming all other parameters are known) 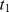 which yields 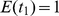. Furthermore, the expression for the number of expected one-nucleotide substitutions between codons 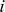 and 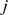, in time 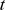, at a site is given by 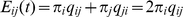 (the simplification is the consequence of time-reversibility). Given two amino-acid residues 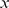 and 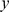 which can be exchanged by a single nucleotide substitution, we can further define 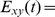 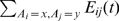, where 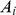 denotes the residue encoded by codon 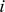. Consider a 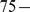
element substitution spectrum vector 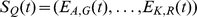, which describes the relative abundance or paucity of a particular type of amino-acid pair substitution under the model defined by 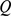. Given two models, 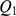 and 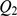, we propose to compare their similarity by computing the distance between the corresponding substitution spectrum vectors evaluated at the corresponding “normalized” times:
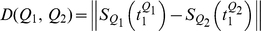
(2)Any norm on the standard 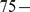
dimension real valued vector space can be used, but for the purposes of this paper we consider the 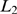 norm, and the corresponding induced Euclidean distance metric.

### Implementation

All models and data sets utilized in this study are implemented as scripts in the HyPhy Batch Language (HBL), and are be available with the current source release of HyPhy [32]. In addition, we have made the GA codon model selector available as an analysis option at http://www.datamonkey.org [46]. The GA model selection code requires an MPI cluster environment with typical runtimes of approximately 36–48 hours for an intermediate-sized alignment (50 taxa) and 32 compute nodes.

## Results

### Power and accuracy analysis on simulated data

Results from both two- and multi-taxon simulations (Table 2, Figure 1) indicated that 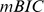 controlled the rates of overfitting, defined as the proportion of replicates that overestimated the number of rate classes 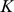, 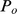. For null (single-rate model) simulations (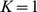), false positive rates were 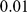 for two-taxon simulations and 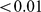 for 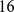-taxon simulation. Neither two- nor multi-taxon simulations showed over-fitting across any simulation scenarios (Table 2). We deliberately designed the procedure to be conservative, since over-fitting is a major concern in statistical model selection. The power to select the correct number of rate classes 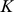 (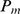) behaved as expected: increasing, and eventually reaching 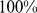, given sufficiently divergent sequences and well resolved rate classes (Table 2). Indeed, the limited information content of alignments where simulated rate classes are similar (i.e 3 rates of 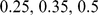), and/or where pairwise sequence divergence is low (0.2), was evident as increased model under-fitting (Table 2), 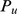. Model under-fitting was substantially reduced when information content was increased, either by boosting the disparity in rate classes, or by elevating sequence divergence and/or number of taxa (Table 2). Further evidence that the GA procedure has high power is provided by the positive association of the difference between 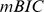 scores of the correct model with 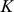 rates, and one with 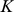-1 rates, and separation between simulated rates, pairwise sequence divergence or number of taxa (Table S1). The ability to assign individual rates to the correct group (as measured by the Rand statistic) was similarly improved, while the variance in numerical rate parameter estimates decreased, for more divergent sequences and rate classes, suggesting that the GA search procedure recaptures most of the rate class structure, given sufficient information.

### Empirical data analysis

We compared the fit of 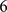 codon substitution models (Table 1) on 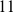 empirical data sets (Table 3), spanning a range of proteins, taxonomic groups and divergence levels, using the BIC to measure goodness-of-fit. Using the GA procedure, we inferred distinct multi-rate models from 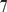 of these data sets (labelled with asterisks in Table 3). The remaining 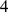 alignments were used for validation such that we could determine the generalizability of two of the GA-fitted models (HIV and IAV) to other alignments from the same taxonomic groups. In 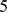 cases, the GA model outperforms every other model (often by a large margin), and in 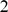 cases it comes in second after the parameter rich REV model (Table 4). Note that the GA model outperforms REV in all 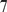 cases under the more conservative 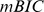 criterion (which was used to inform the GA). Data set specific GA models consistently fit the data better than state-of-the-art empirical (ECM) and mechanistic (LCAP) models.

**Table 4:** Comparison of empirical model fits using BIC.

	S+F61	ECM+F61	ECM+F61+ 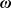	LCAP+F61	GA 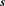 +F61	REV+F61
ATP cone*	42176.4 (5)	41563.4 (3)	41329.6 (2)	49049 (6)	**41214.6 (1)**	41831.6 (4)
NADH5 C*	69057.9 (3)	69148.1 (5)	69099 (4)	72329.4 (6)	68086.3 (2)	**67211.8 (1)**
Transketolase C*	63509.4 (5)	61436.2 (2)	61443.7 (3)	67819.7 (6)	**61227.8 (1)**	61469.4 (4)
Rhodopsin *	27918.7 (5)	28583.3 (6)	27769.6 (3)	27614.7 (2)	**27322.7 (1)**	27781.3 (4)
Yeast Protein YAL038W*	21219.1 (5)	22246.1 (6)	20988.8 (2)	21098.2 (3)	**20822.7 (1)**	21142.7 (4)
HIV-1 *pol* Group M*	148650 (4)	158788 (6)	156792 (5)	146381 (3)	145338 (2)	**145209 (1)**
HIV-1 *pol* subtype B	113583 (4)	119721 (6)	119196 (5)	111249 (3)	**108251 (1)**	110113 (2)
HIV-1 *pol* subtype C	127143 (4)	134719 (6)	133794 (5)	125407 (3)	124434 (2)	**123346 (1)**
Influenza A *HA* *	17803.9 (3)	19479.7 (6)	18883.3 (5)	17750.6 (2)	**17558.8 (1)**	18110.3 (4)
Influenza A *HA* H5N1	**28326.2 (1)**	28987.1 (6)	28911.7 (5)	28382.8 (3)	28347.2 (2)	28904.2 (4)
Influenza A *HA* H3N2	**7527.03 (1)**	7649.29 (4)	7658.29 (5)	7562.29 (3)	7546.24 (2)	8096.39 (6)

The best model (with smallest BIC) is shown in boldface and the rank of each model is provided in parentheses.

*Reference alignments from which GA models were estimated.

**Figure 2:**
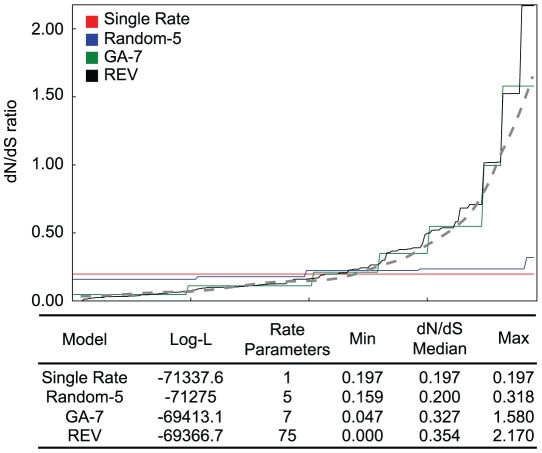
Evolutionary rate estimation as “curve fitting.” An example from HIV-1 polymerase gene alignment for which the 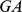 inferred 7 non-synonymous rate classes. The idealized biological rate distribution (unobservable) is depicted by the dashed line. The goodness of fit, the complexity of the models, and the range of maximum likelihood parameter estimates are listed in the table.

An intuitive understanding of the model selection process via the GA may be gained by thinking of it as a non-linear curve fitting problem, where the “true” curve is the unobserved distribution of biological substitution rates (Figure 2). We consider the 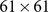 substitution rate matrix for a codon model, extract non-synonymous rates for the 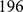 above-diagonal entries which correspond to one-step non-synonymous substitutions and rank them in an increasing order to obtain monotonically increasing rate curves as shown in (Figure 2). Note that because the ratios for all substitutions between the same pair of amino-acids (of which there are 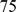 pairs) are identical, this will create steps in such curves. In the case of one non-synonymous substitution rate (SR) the curve is a flat line at the estimated average non-synonymous substitution rate across all residue pairs. This is easily improved on by a random model which assigns non-synonymous substitutions randomly to one of 5 rate classes. At the other extreme lies the general time reversible models with 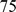 estimated rates. Since we have no *a priori* reason to believe that any two non-synonymous substitution rates will be exactly the same, REV is the most biologically realistic of the models which assume time-reversibility and only single nucleotide substitutions. However, fitting the parameter rich REV model to limited data is statistically unsound. The GA-approach, instead, searches for the best (in an information theoretic sense) step-wise smoothing of the biological distribution given the data available (Figure 2).

The “generalist” ECM model sacrifices gene-level resolution, in some cases so dramatically that it underperforms the single-rate model, even with the correction factor 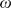 (Table 4). For instance, ECM appears to be ill suited for the analysis of viral genes. LCAP, on the other hand, performs poorly for highly divergent data sets; indeed the original validation of LCAP took place on relatively closely related yeast species [18], and the mechanistic properties assumed by the model may be insufficient in alignments spanning multiple genera and taxonomic groups. To test whether GA structured models are generalizable, we estimated two viral models: one for HIV-1 polymerase and one for human IAV hemagglutinin. We then applied each of these models (holding the inferred class structure fixed) to two additional samples of sequences from the same gene, obtained independently from the training sample. In all 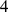 cases 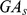 outperformed ECM, ECM+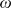 and LCAP by wide margins, lending credence to the claim that data-driven structured models recover substitutional biases that are shared by other samples shaped by similar evolutionary parameters. Curiously, for very low divergence (and low information content) intra-serotype IAV alignments, the single rate model was preferred to all other models by BIC, suggesting that there are biologically interesting alignments, which do not contain sufficient amino-acid variability to indicate the use of a multi-rate model.

**Figure 3:**
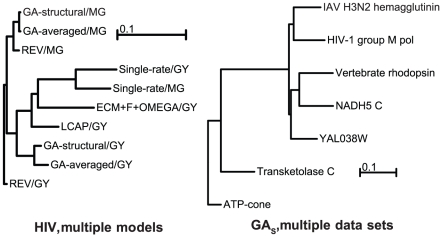
Neighbor-joining [57] trees built from matrices of pairwise substitution spectrum distances (**Eq. 2**) computed between different models fitted to the HIV-1 group M *pol* alignment, and between 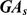 models inferred from different alignments.

As a test of protein-specificity of 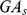 models, we randomly selected four Pandit data sets to assess how well 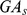 models inferred from unrelated proteins fitted these data (Table S2). Not surprisingly, ECM was the best model in 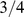 cases, because it was derived as the best “average” protein model. LCAP topped the list in one case, but placed outside the top three in the other three cases. The GA structured models, being tailored to specific proteins, tended to differ from each other (Table S3) and did not perform well on proteins from different families. However, the GA structured models for ATP cone and Transketolase C did outperform the LCAP model in 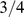 cases, which suggests some similarity between the respective protein families in those cases. This indicates the GA models fitted to different proteins may be generalizable, with the degree limited by taxonomy, protein function or both. The generalizability of GA models could further be quantified by evolutionary fingerprinting of genes [27]; see also Figure 3(b).

### Further analysis of GA multi-rate models

A GA search run typically examines between two- and a hundred-thousand potential models, e.g. 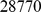 models with 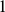 to 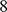 rate classes for the HIV-1 group M *pol* dataset. 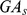, which we compared to existing models in the previous section, is simply the single “best” model, i.e. the model that minimized the 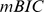 criterion among all those examined during the run. Further, we estimate the credible set of models as those models whose evidence ratio versus the best model is sufficiently large (see methods). Among 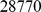 models fitted to HIV-1 pol by the GA, 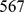 belonged to the credible set. Given sufficient data and knowing that the true model is in the set examined by the GA, e.g. in the long 2-sequence simulations discussed above, the size of the credible set frequently shrinks to 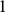 (the true model). These structured (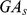) and model-averaged (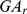) models can be analyzed further to draw inferences of the substitution process.

**Figure 4:**
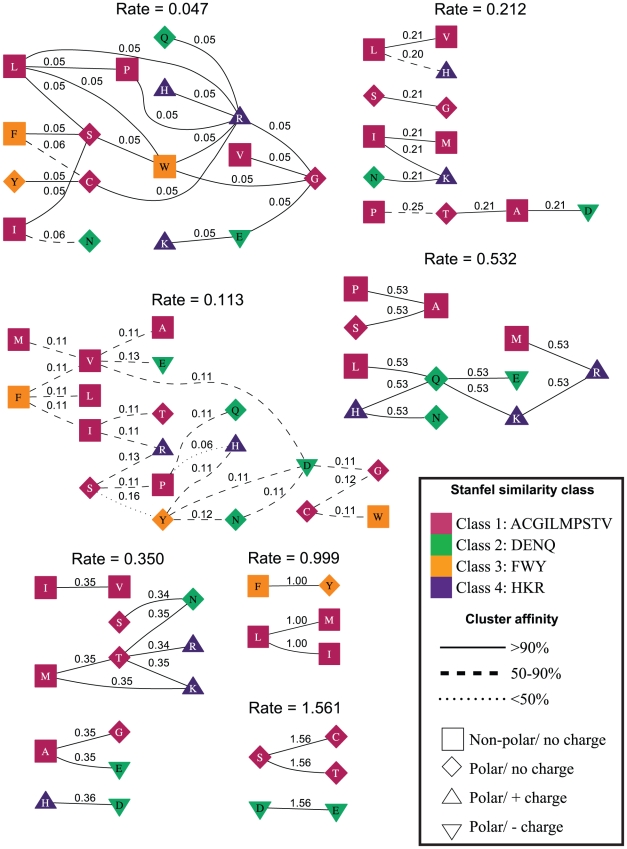
Evolutionary rate clusters in structured GA models (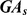) inferred from the HIV-1 group M *pol* alignment. Each cluster is labeled with the maximum likelihood estimate of its rate inferred under 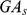. The residues (nodes) are annotated by their biochemical properties and Stanfel class, and the rates (edges) are labeled with model-averaged (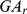) rate estimates. The style of an edge is determined by its cluster affinity, where high cluster affinities indicate that a large proportion of models in the credible set were consistent with the structured 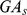 model.

**Figure 5:**
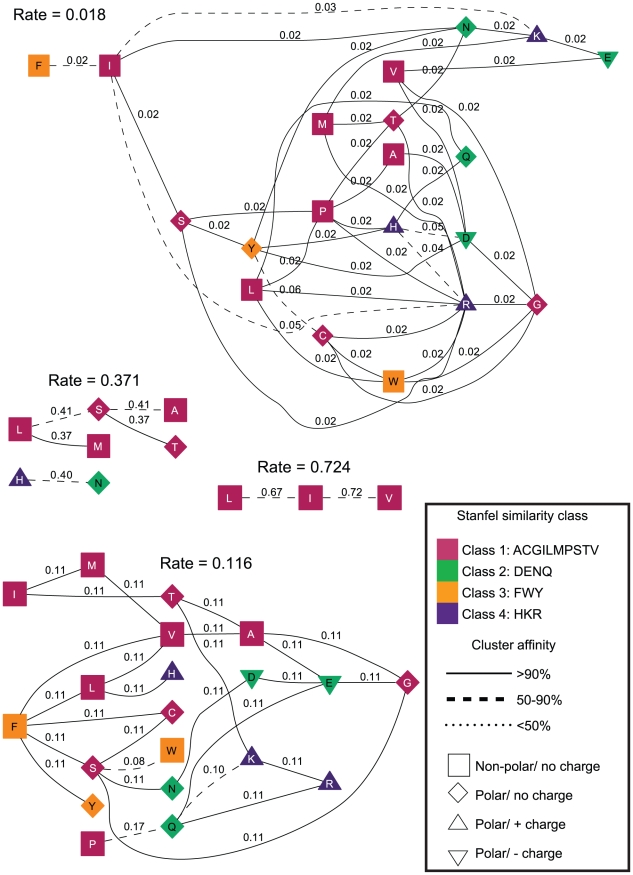
Evolutionary rate clusters in structured GA models (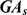) inferred from the vertebrate rhodopsin protein alignment. Each cluster is labeled with the maximum likelihood estimate of its rate inferred under 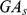. The residues (nodes) are annotated by their biochemical properties and Stanfel class, and the rates (edges) are labeled with model-averaged (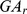) rate estimates. The style of an edge is determined by its cluster affinity, where high cluster affinities indicate that a large proportion of models in the credible set were consistent with the structured 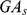 model.

For instance, the structured 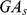 model identifies which residue pairs are exchanged rarely, relative to the baseline synonymous rate. In Figures 4 and 5 we cluster the pairs of residues which have the same rate of non-synonymous substitution; residues are labelled by Stanfel class and physicochemical properties. Note that the same residue can be present as a node in multiple clusters because the GA partitions residue pairs (i.e. the rates between them), not the residues themselves. The model reveals a startling heterogeneity of substitution rates in HIV-1 *pol*: the single rate 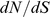 estimate of 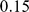 is resolved into 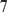 rate classes (Figure 4), with relative non-synonymous substitution rates ranging from 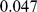 (20 residue pairs) to 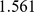 (3 residue pairs); a similar range is revealed for other datasets (Table 3). It is remarkable that some of the non-synonymous substitutions occur at rates matching or exceeding the gene-average rate of synonymous substitutions. This can be interpreted, for instance, as lack of selective constraint on particular residue substitutions gene-wide, or evidence of directional selection when some residues are preferentially replaced with others. Regardless of how this result is interpreted, a remarkable complexity of substitution patterns is revealed by the analysis. We hypothesize that such patterns reflect complex dynamics of substitutional preferences that may be shared by multiple samples of the same genes. This hypothesis is supported (by the goodness-of-fit of 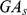 vs other models) on HIV-1 and IAV samples in this study (Table 4), and we are currently undertaking the GA analysis of several thousand alignments to confirm this finding.

One of the benefits of using the 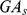 model instead of REV or other models is that the former model automatically classifies all substitutions into similarity groups, supplying a data-driven analog of “conservative” or “radical” substitutions, previously defined *a priori* based on chemical properties of the residues, or a more sophisticated multi-property basis defined in the LCAP model. For example, the 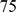 substitution rates are partitioned into seven classes in the 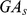 model inferred from HIV-1 *pol*, and into 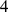 rate classes for the 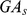 model fitted to a smaller, but more divergent vertebrate rhodopsin alignment (Figure 5).

Multi-model inference is instrumental in assessing how robust the clustering assignment made by 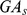 is. In Figures 4 and 5, we present this information by labeling individual substitution rates with their model averaged values. An examination of the numerical differences between rate estimates (for a particular amino-acid pair) obtained under 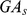 and 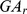 can reveal ambiguities in assigning a particular rate to a class. More formally, we can compute a model averaged support for the probability that rates 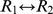 and 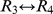 (for residues 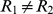, 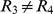) are in the same class, as described above, or that the corresponding edges 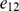 and 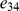 are in the same component of the rate graph (Figure 4). If 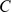 is a cluster defined by 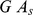 (with the number of nodes in 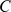, 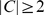), we define the *cluster affinity* of an edge 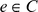 as the mean of the model averaged estimates of the probabilities that edge 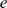 and other edges in 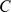 belong to the same cluster:
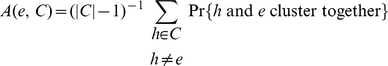
If 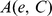 is below a certain threshold, for instance 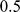 for majority rule, then cluster membership of edge 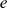 is ambiguous. For example, the 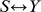 substitution pair with a model-averaged non-synonymous rate of 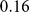 is one of two rate pairs with low (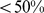) cluster affinity for HIV-1 (Figure 4). Two of the inferred 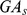 rate classes have non-synonymous rates of 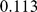 and 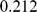, respectively, and the placement of model-averaged rate for 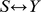 between the two values is indicative of the alternate assignment of this substitution pair to these two rate classes among models of the credible set. A larger training data set may be able to infer an additional intermediate rate class between 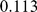 and 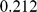. While 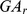 yields more robust numeric estimates of substitution rates for a single data set, 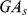 has better 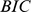 fit on validation HIV and IAV alignments (results not shown).

### The relationship between substitution rates and residue properties

**Figure 6:**
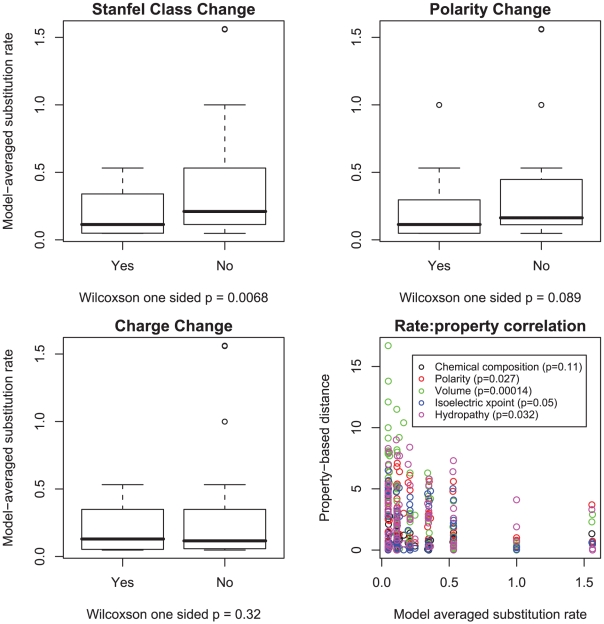
Correlations of lower substitution rates and property preservation in the HIV-1 group M *pol* alignment. Model-averaged 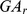 rates were stratified by whether or not they involved a change in polarity, charge or Stanfel class, the medians of two rate distributions were compared using a one sided Wilcoxon rank-sum test. We further correlated the magnitude of substitution rates with one of five property-based distances between the corresponding residues (defined in [18]) using a one-sided (negative correlation) Pearson product-moment correlation test.

**Figure 7:**
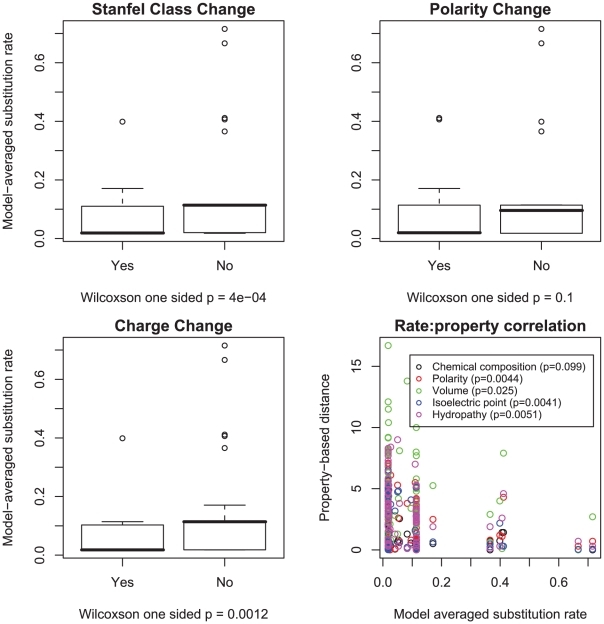
Correlations of lower substitution rates and property preservation in the vertebrate rhodopsin alignment. Model-averaged 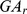 rates were stratified by whether or not they involved a change in polarity, charge or Stanfel class, the medians of two rate distributions were compared using a one sided Wilcoxon rank-sum test. We further correlated the magnitude of substitution rates with one of five property-based distances between the corresponding residues (defined in [18]) using a one-sided (negative correlation) Pearson product-moment correlation test.

The expectation that substitutions which preserve amino acid physicochemical properties occur at a lower rate than property-altering substitutions has previously been evaluated in the maximum likelihood codon model context [20], [47]. However, in published work, property-altering and property-conserving amino acid classes are defined *a priori*, whereas in the GA approach amino acid substitution pairs are first partitioned into classes based on rate similarity, and thereafter property preserving versus property-altering rates can be compared. The increased substitution rate of property preserving substitutions, holds largely – but not universally – for 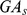 and 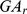 rates, as evidenced in Figures 6 and 7. For example, in the vertebrate rhodopsin sample, the median rate of charge-changing substitutions is significantly lower than the charge-preserving substitutions, but the two medians are not significantly different in the HIV-1 *pol* sample. The rates were negatively correlated (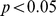, one-sided Pearson product moment test, no multiple test correction) with 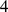 out of 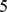 property-based distances (polarity, volume, isoelectric point and hydropathy) that form the basis of the LCAP model. However, while the broad pattern follows the expectation, the consistently better fit of 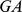-based models, and the presence of strong outliers, such as 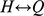 and 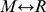 in the 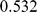 cluster of HIV-1 rates (Figure 4), suggests that our data driven approach detects significant deviations from purely biochemical rate expectation. These deviations could be attributed to selective pressures which promote property changes, or could arise because not all biologically relevant important properties have been included into structured models.

One benefit of our approach over the “amino acid class” models [20], [47] is that transitivity of rates (i.e. the requirement that if 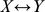, and 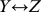 are in the same rate class, then so is 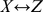) is not enforced by the GA models. Because we focus on modeling single-nucleotide substitution rates only, the structure of the genetic code itself contradicts transitivity. For instance both 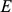 (encoded by 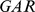) 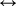 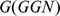 and 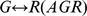 are one-step substitutions, but 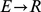 is not. Further, since amino acid class models only estimate two non-synonymous rates (within and between classes), it is a necessary condition that non-synonymous rates which change amino acid property be shared irrespective of how much the property is being changed. For instance, substitution rates which change charge from negative to positive will be the same as those which change charge from negative/positive to uncharged. If amino acid substitutions that result in a positive charge are favored, then these transitive conditions are not representative of the substitution process. Furthermore, the amino acid class models assume all substitutions within classes occur at the same rate. This is a very strong assumption since some amino acids with the same physicochemical property class are separated by more than one nucleotide substitution, e.g. positively charged amino acids 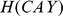 and 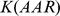. Although we do not account for multiple nucleotide substitutions in the GA model directly (but see below), previous work has demonstrated that these occur at lower rates than single-nucleotide substitutions [9], [10], [48].

### Model clustering

Using the substitution spectrum distance defined in Equation (2), it is easy to construct a hierarchical clustering of several models fitted to the same dataset, as well as between models fitted to different datasets. The former is useful to interpret how much difference in predicted substitution patterns over a unit of evolutionary time there is between different descriptions of the same data, whilst the latter naturally extends the concept of evolutionary fingerprinting of non-homologous genes [27]. For HIV-1 pol (Figure 3), 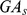 and 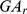 models both clustered closely with the rate substitution pattern predicted by the REV model, followed by LCAP, ECM+F+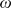, and finally – distant single rate models. The similarity between REV and GA models was especially strong for the 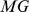 parameterization, under which the GA models were inferred. In a between-genes model comparison (Figure 3), the two viral alignments clustered together, as did the two most divergent alignments (ATP-cone and Transketolase C).

### Effects of substitution models on statistical inference

Statistical inference procedures based on phylogenetic models have varying degrees of robustness with respect to the substitution rate matrix used in the analysis. For a multi-rate model, it is intuitively clear that the types of inference that rely on “mean” rates should be minimally affected, whereas those that depend on the individual residue rates can be affected significantly. We examine several such measures inferred from two of the datasets in this study.

Branch length estimates are essentially unchanged when moving from the single-rate (SR) model to a 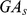 model. On the example HIV-1 *pol* dataset, the total tree length changed from to 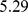 (SR) expected substitutions/nucleotide to 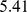 (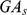), and the lengths of individual branches were nearly perfectly linearly correlated with linear regression slope of 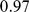, intercept of 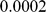 and 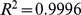.

Ancestral character reconstruction is considerably more sensitive to the substitution model. In the vertebrate rhodopsin data set, for example, the joint maximum likelihood ancestral reconstruction [49] under SR and 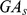 models differed in the number of inferred non-synonymous substitutions at 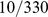 sites, with more non-synonymous substitutions in 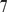 cases under 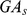. At 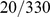 sites substitutions were mapped to a different set of branches.

Site-specific diversifying selection screens are likely to be profoundly affected by a switch from single- to multi-rate models. Consider the FEL method [50], where the SR model is fitted site-by-site and a likelihood ratio test (LRT) is used to test whether 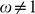. First, because 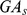 defines multiple substitution classes, one can now apply a variety of tests to see *which* non-synonymous rates at a given site exceed the baseline synonymous rate. To explore this approach for a 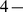
rate 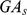 multi-class model applied to the vertebrate rhodopsin alignment, we performed 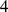 LRT tests, where we independently constrained each non-synonymous rate parameter (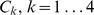, Table 1) to be equal to 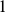 at a site (neutral evolution in class 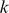), vs an unconstrained 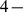
parameter alternative. This is analogous to performing a test for selection at a site by constraining the non-synonymous rate to be equal to the synonymous rate, and comparing the fit to the unconstrained model (FEL), except that we only place the constraint on one rate class at a time. At 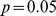, the standard (SR) FEL reported 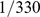 (codon 54) sites as being under diversifying selection (positively selected). However, for the 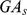 model, there were 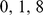 and 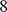 positively selected sites for the four substitution classes (Figure 5, increasing rate magnitude), respectively at the Bonferroni corrected 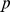 of 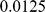. Codon 54 was selected only with the fastest rate class (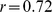), because the signal of selection is driven by a large number of 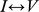 substitutions. Only one codon (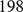) was selected with two or more different tests (rate classes 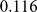 and 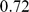).

### The effect of site-to-site rate variation

We remark that the effects of site-to-site rate variation and multiple non-synonymous rates appear to be largely additive, and not confounded. This is a critical observation: if the effects are confounded, then we cannot justify inferring the multi-rate model independently assuming no site-to-site rate variation, as is done in this manuscript for computational expedience. To illustrate, we fitted both a constant rate model and the general bivariate distribution [27], with and without accounting for multiple non-synonymous rate classes (Table 5). The constant rate model assumes all sites share the same rate of substitution, whereas a general bivariate distribution infers the number of site-to-site variation classes from the data [27]. These models were fitted to the vertebrate rhodopsin alignment, which exhibits extensive site to site rate heterogeneity. The 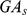 inferred 4 non-synonymous rate classes for the rhodopsin alignment, whereas the single 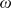 has one, resulting in three degrees of freedom for the comparison of these models. When the general bivariate model was fitted with a single 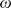 or 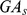, 6 and 7 site classes were inferred, respectively, resulting in 4 degrees of freedom for the comparison of single 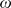 and 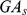 models (3 rate and 1 site class are added to the 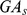 model). The important observation is that the addition of site-to-site rate variation component resulted in a significant improvement in log likelihood scores, regardless of the underlying substitution model (single 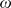 or 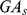). This suggests that by allowing multiple rate classes, we are not merely fitting variability in site-to-site selective constraints. However, as the cost of computing cores in clusters decreases, we expect that it will become practical to infer 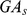 models with the site-to-site rate variation component included directly in the search procedure.

**Table 5:** The effects of modeling site-to-site rate variation and multiple non-synonymous rates in the vertebrate rhodopsin alignment using the MG frequency parameterization.

	Single 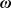	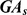 (+3 df)	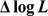
Constant rates	−13382.6	−12954.2	428.4
General bivariate rates (+4 df)	−12780.8	−12500.4	280.4
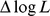	601.8	453.8	

The entry for joint effect was obtained by running the general bivariate model fit using the 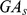 model obtained under the assumption of constant site-to-site rates. df = degrees of freedom.

### The effect of allowing multiple instantaneous nucleotide substitutions

Recent extensions of codon models which permit multiple instantaneous nucleotide substitutions [9], [10], [48] tend to fit the data better than their traditionally parameterized counterparts. We explored whether this observation held for 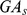 models using a straightforward extension of the rate matrix in Equation (1), following the ideas of [9]. We introduce four new independently estimated parameters to model the relative rates of synonymous (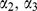) and non-synonymous (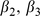) substitutions which replace two or three nucleotides, and modulate them by the product of the corresponding nucleotide rates 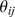 and the target codon frequency 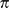 (assuming the GY parameterization with the F61 estimator). For instance the rate of synonymous substitution (Serine) from 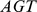 to 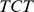 is 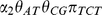, while the rate of non-synonymous substitution 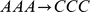 (Lysine to Proline) is 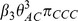.

Table 6 summarizes the effect of adding multi-step substitutions to SR and 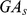 models for the vertebrate rhodopsin alignment. Much as was the case for site-to-site rate variation, the effects of multiple single-step non-synonymous rates and the non-zero rates of two or three nucleotide substitutions are additive at the 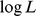 level, and the estimates of single-step substitution rates were minimally influenced by the presence of the multi-step component (results not shown). The 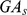 model augmented to allow multi-step substitutions can be directly compared to the Mechanistic-Empirical codon (MEC) model [9] coupled with the LG [51]empirical amino-acid substitution model (selected as the best fitting empirical model using the procedure implemented on http://www.datamonkey.org. Assuming no site-to-site rate variation, BIC of the MEC model is 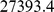, while that of the 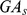
+multi-step model using the HKY85 nucleotide component (a direct analog to the MEC model) is 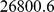, once again highlighting how strongly the substitution process in an individual gene appears to deviate from the “average” encoded by empirical protein models.

**Table 6:** The effects of modeling multi-nucleotide instantaneous substitutions and multiple non-synonymous rates in the vertebrate rhodopsin alignment using the F61 frequency parameterization.

	Single 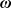	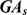 (+3 df)	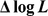
Single-nucleotide substitutions only	−13317.6	−13005.5	312.1
Single and multi-nucleotide substitutions (+4 df)	−13033.4	−12712.5	320.9
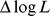	284.2	293	

The entry for joint effect was obtained by augmenting the 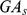 model with non-zero rates for substitutions requiring two or three nucleotide changes. df = degrees of freedom.

The GA could be modified to search for optimal partitions among all 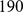 pairs of rates, for example using the above parameterization, but as the rhodopsin example indicates, the single-step and multi-step rate rate components appear to be effectively independent. We will explore this option in future versions of the model selection GA.

## Discussion

In this manuscript we have developed, validated and benchmarked a procedure to quickly and reliably infer a multi-rate model from the combinatorially large class of general time-reversible codon substitution models. Using extensive simulations, we demonstrated that our conservative 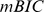 model selection criterion controls over-fitting and has excellent power on data sets of biologically realistic size, inferring the exact model simulated given sufficient sequence divergence and length. We have previously argued against using the single rate model as a benchmark against which multi-rate models should be compared, since it is trivial to improve upon using a random assignment of substitutions to rate classes [19]. We reiterate this argument here, and suggest we should rather consider how well a multi-rate model approximates the REV model (Figure 2), given the limitations posed by the information content in an alignment. On a diverse collection of biological data, 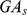 models consistently outperform the best-in-class empirical and mechanistic models, and match the performance of fully parameterized general time reversible models with only a few biologically relevant rate parameters (Table 4). Therefore, the 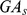 provides goodness of fit matching or exceeding that of REV, with substantially fewer parameters and is thus computationally and statistically feasible for downstream analyses.

ModelTest [22] has been universally adopted to mitigate the effect of model misspecification on statistical inference from nucleotide data, and we posit that a robust codon model selection procedure, for example the one offered in this paper, will play a similar role for codon data. In the same vein as ModelTest, we infer the best model (which we term the 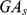) for an alignment, and also utilize model averaging [26] to achieve more robust estimates of biologically relevant parameters. Certain applications of codon models, such as divergence estimation, appear unaffected by the gross biological over-simplification of single-rate models, because they are only influenced by the mean of substitution rates. Others, including ancestral sequence reconstruction (e.g. for guided site directed mutagenesis, [38]), substitution mapping (e.g. for co-evolutionary analysis, [52]) and character sampling (e.g. for data augmentation modeling approaches, [53]) can see moderate effects. Applications which are tightly integrated with the substitution model and the interpretation of its parameters, such as site-by-site positive selection detection (e.g. [50], [54]), will be profoundly affected by the introduction of multiple rates. Our results strongly argue against the prospect of deriving a single “generalist” model of codon evolution, that is capable of fitting most protein alignments well. Hence we should strive to fit both gene and taxonomy specific models of codon evolution. We further hypothesize that independent alignments representing a gene or a protein family will share most of the model structure and confirm this with HIV-1 polymerase and Influenza A virus hemagglutinin examples. While significant further validation is required and is currently underway, we assert that a collection of substitution models inferred from carefully selected training datasets can provide a useful library of organism and gene-specific models to be used in inference on codon sequences. This is conceptually similar to a library of Hidden Markov profile models, inferred from seed alignments, used for detecting protein domain homology in the Pfam database [55]. In order to facilitate the process of generating gene and taxonomic specific multi-rate codon models we have implemented the GA on our free analysis webserver (http://www.datamonkey.org, [46]), and have begun to assemble a library of representative multi-rate substitution models that are needed to reduce biases in those procedures that are sensitive to model misspecification.

The inference of the multi-rate codon models should be considered more than just a necessary step for downstream applications. By examining the structure of inferred rate classes, we argue that the 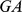 captures the *a priori* expectation that radical changes in one or more biochemical properties of a residue happen relatively infrequently, but also that a mere reliance on such data-abstract mechanistic properties misses out important gene and organism specific peculiarities of the evolutionary process. For instance the elevation of substitution rates between amino acids that do not preserve physicochemical properties may be indicative of selective pressures which promote property changes. These selective pressures are of crucial importance in understanding evolution in viruses, such as HIV-1, known to evade host immune response [56]. We anticipate that considering specific substitution types when estimating selective pressures will improve power, as demonstrated with our multi-rate FEL analysis of vertebrate rhodopsin. However, this may also increase the rate of false positives, a conjecture that can be evaluated with straightforward, but laborious simulations.

Finally, we demonstrate how simple metrics on 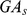 models inferred from different (e.g. non-homologous) alignments can be used to obtain an objective measure of similarity and disparity in substitutional preferences in different proteins and thus improve the resolution in evolutionary fingerprinting of genes [27].

## Supporting Information

Table S1 Difference in mean (standard deviation) model *mBIC* scores for multi-taxon simulations. *D* is the average pairwise divergence; *mBIC_n_* is the difference in model *mBIC* score between the model with *n*−1 rates and a more complex with *n* rates; P is the proportion of correctly identified models for 100 simulations. Positive *mBIC* scores indicate preference for the more complex model with *n* rates, i.e. *mBIC_n_* = *mBIC_n−1_−mBIC_n_*.
(0.04 MB PDF)

Table S2 Randomly selected Pandit data model comparisons using BIC. In each case we fitted the ECM, LCAP and *GA_s_* models to each of four randomly selected Pandit datasets. Model ranks (BIC/difference in BIC score relative to the best model) are shown.
(0.03 MB PDF)

Table S3 Qualitative comparison of structured GA models.
(0.02 MB PDF)
