# Supplementary material for: CodonTest: Modeling Amino Acid Substitution Preferences in Coding Sequences
Source: PLoS Comput Biol. 2010 Aug 19;6(8):e1000885. doi: 10.1371/journal.pcbi.1000885 (PMC2924240; doi:10.1371/journal.pcbi.1000885)
Supplement: Table S1 — Difference in mean (standard deviation) model mBIC scores for multi-taxon simulations. D is the average pairwise divergence; mBICn is the difference in model mBIC score between the model with n−1 rates and a more complex with n rates; P is the proportion of correctly identified models for 100 simulations. Positive mBIC scores indicate preference for the more complex model with n rates, i.e. mBICn = mBICn−1−mBICn. (0.04 MB PDF) [file pcbi.1000885.s001.pdf]

Table S1. Difference in mean (standard deviation) model $mBIC$ scores for multi-taxon simulations. D is the average pairwise divergence; $mBIC_n$ is the difference in model $mBIC$ score between the model with $n-1$ rates and a more complex with n rates; P is the proportion of correctly identified models for 100 simulations. Positive $mBIC$ scores indicate preference for the more complex model with n rates, i.e. $mBIC_n = mBIC_{n-1} - mBIC_n$.

simulation	num taxa	D	$mBIC_2$	$mBIC_3$	$mBIC_4$	P
single rate	16	0.2	-44.01 (4.22)	n/a	n/a	1.00
(0.25, 1.0)	16	0.2	345.12 (37.45)	-28.66 (3.29)	n/a	1.00
(0.25, 0.5, 1.0)	16	0.2	195.38 (32.0)	-19.233 (7.94)	-0.28 (2.76)	0.23
(0.25, 0.75, 1.5)	16	0.2	426.21 (33.86)	94.70 (18.30)	-13.61 (2.46)	1.00
(0.25, 0.5, 1.0)	16	0.5	516.08 (46.39)	90.28 (18.28)	-13.80 (2.17)	1.00
(0.25, 0.5, 1.0)	32	0.2	378.84 (42.03)	54.04 (26.96)	-13.08 (5.48)	0.89
