# Supplementary material for: CodonTest: Modeling Amino Acid Substitution Preferences in Coding Sequences
Source: PLoS Comput Biol. 2010 Aug 19;6(8):e1000885. doi: 10.1371/journal.pcbi.1000885 (PMC2924240; doi:10.1371/journal.pcbi.1000885)
Supplement: Table S2 — Randomly selected Pandit data model comparisons using BIC. In each case we fitted the ECM, LCAP and GAs models to each of four randomly selected Pandit datasets. Model ranks (BIC/difference in BIC score relative to the best model) are shown. (0.03 MB PDF) [file pcbi.1000885.s002.pdf]

Table S2. Randomly selected Pandit data model comparisons using BIC. In each case we fitted the ECM, LCAP and GA_s models to each of four randomly selected Pandit datasets. Model ranks (BIC/difference in BIC score relative to the best model) are shown.

Source<br>Description	Pandit/Pfam PF00207<br>Alpha-2-macroglobulin	Pandit/Pfam PF01486<br>K-box region	Pandit/Pfam PF00829<br>Ribosomal prokaryotic L21	Pandit/Pfam PF05697<br>Bacterial trigger factor (TF)
ECM+F61	1 (33080.8)	5 (811.9)	1 (35613.6)	1 (47405)
LCAP+F61	7 (321.9)	1 (35896.4)	4 (893.4)	6 (1687.2)
GA_s ATP cone	2 (29.8)	4 (710)	2 (624.3)	3 (1120.5)
GA_s NADH5 C	8 (347.9)	2 (257.3)	6 (1072.1)	5 (1438.9)
GA_s Transketolase C	3 (80.8)	7 (961)	3 (695.1)	2 (772.8)
GA_s Rhodopsin	5 (211.1)	3 (605.4)	5 (932.8)	4 (1326)
GA_s YAL038W	6 (254.2)	8 (1083.9)	8 (1238.8)	7 (1712.3)
GA_s HIV-1	4 (181.4)	6 (937.6)	7 (1130.8)	8 (1834.6)
GA_s IAV HA	9 (407.1)	9 (1119.7)	9 (1393.6)	9 (1941.3)
