# Supplementary material for: CodonTest: Modeling Amino Acid Substitution Preferences in Coding Sequences
Source: PLoS Comput Biol. 2010 Aug 19;6(8):e1000885. doi: 10.1371/journal.pcbi.1000885 (PMC2924240; doi:10.1371/journal.pcbi.1000885)
Supplement: Table S3 — Qualitative comparison of structured GA models. (0.02 MB PDF) [file pcbi.1000885.s003.pdf]

Table S3. Qualitative comparison of structured GA models

model 1	model 2	Rand statistic	Mean Random Rand	P
ATP cone	NADH5 C	0.584	0.618	1
ATP cone	Transketolase C	0.709	0.657	1
ATP cone	Yeast YAL038W	0.618	0.618	0.448
ATP cone	HIV-1 *pol*	0.674	0.668	0.178
ATP cone	IAV HA	0.563	0.578	0.969
ATP cone	Rhodopsin	0.565	0.618	1
NADH5 C	Transketolase C	0.617	0.626	0.903
NADH5 C	Yeast YAL038W	0.585	0.595	0.893
NADH5 C	HIV-1 *pol*	0.642	0.663	0.986
NADH5 C	IAV HA	0.560	0.577	0.997
NADH5 C	Rhodopsin	0.571	0.594	1
Transketolase C	Yeast YAL038W	0.610	0.652	1
Transketolase C	HIV-1 *pol*	0.707	0.717	0.916
Transketolase C	IAV HA	0.601	0.603	0.534
Transketolase C	Rhodopsin	0.558	0.652	1
Yeast YAL038W	HIV-1 *pol*	0.635	0.663	0.995
Yeast YAL038W	IAV HA	0.535	0.577	1
Yeast YAL038W	Rhodopsin	0.604	0.573	0.005
HIV-1 *pol*	IAV HA	0.614	0.609	0.248
HIV-1 *pol*	Rhodopsin	0.568	0.663	1
